# Kinematic–Muscular Synergies Describe Human Locomotion with a Set of Functional Synergies

**DOI:** 10.3390/biomimetics9100619

**Published:** 2024-10-13

**Authors:** Valentina Lanzani, Cristina Brambilla, Alessandro Scano

**Affiliations:** Advanced Methods for Biomedical Signal and Image Processing Laboratory, Institute of Intelligent Industrial Systems and Technologies for Advanced Manufacturing (STIIMA), Italian Council of National Research (CNR), 20133 Milan, Italy; valentina.lanzani@stiima.cnr.it (V.L.); alessandro.scano@stiima.cnr.it (A.S.)

**Keywords:** mixed matrix factorization, kinematic–muscular synergies, EMG, gait, muscle synergies, motor control, kinematics

## Abstract

Kinematics, kinetics and biomechanics of human gait are widely investigated fields of research. The biomechanics of locomotion have been described as characterizing muscle activations and synergistic control, i.e., spatial and temporal patterns of coordinated muscle groups and joints. Both kinematic synergies and muscle synergies have been extracted from locomotion data, showing that in healthy people four–five synergies underlie human locomotion; such synergies are, in general, robust across subjects and might be altered by pathological gait, depending on the severity of the impairment. In this work, for the first time, we apply the mixed matrix factorization algorithm to the locomotion data of 15 healthy participants to extract hybrid kinematic–muscle synergies and show that they allow us to directly link task space variables (i.e., kinematics) to the neural structure of muscle synergies. We show that kinematic–muscle synergies can describe the biomechanics of motion to a better extent than muscle synergies or kinematic synergies alone. Moreover, this study shows that at a functional level, modular control of the lower limb during locomotion is based on an increased number of functional synergies with respect to standard muscle synergies and accounts for different biomechanical roles that each synergy may have within the movement. Kinematic–muscular synergies may have impact in future work for a deeper understanding of modular control and neuro-motor recovery in the medical and rehabilitation fields, as they associate neural and task space variables in the same factorization. Applications include the evaluation of post-stroke, Parkinson’s disease and cerebral palsy patients, and for the design and development of robotic devices and exoskeletons during walking.

## 1. Introduction

Human locomotion is the result of a coordinated action of a large number of muscles. The number of muscles largely exceeds the number of degrees of freedom and, hence, the musculoskeletal system is highly redundant [1]. An accepted theory of motor control suggests that the central nervous system (CNS) produces movement through a combination of a limited number of spatial and/or temporal patterns, referred to as muscle synergies [2]. Muscle synergies can be extracted through the decomposition of electromyographic (EMG) signals in spatial [3], temporal [4], spatiotemporal [5] or space-by-time [6] motor modules. The spatial synergy model, which is the most used, describes the generation of motor commands as the combination of task-independent muscle weights (synergies) and task-dependent temporal activations.

So far, human gait has been analyzed in the framework of synergistic control in the muscle and in the kinematic domains separately. Studies agree in finding four to five motor synergies as fundamental blocks underlying locomotion, corresponding to biomechanical functions, such as weight acceptance, push off, swing, and leg deceleration [4,7,8,9]. In particular, the first two synergies and the last synergy (in chronological order with respect to a stride) are usually confirmed by most of the literature: weight acceptance at heel strike is mainly characterized by the activity of glutei muscles, tensor fascia latae, vastus medialis and rectus femoris, to guarantee the stabilization of the joints during weight transfer; the push off synergy is represented by the muscles needed to provide propulsion, i.e., gastrocnemii, soleus and peroneus longus; at the end of the swing phase, leg deceleration is controlled by the hamstring muscles [4,7,8,9]. In the first part of the swing phase, one or two synergies are usually identified, in which the trunk muscles are also involved. If only one synergy is considered, the main muscles involved are trunk muscles, rectus femoris and tibialis anterior [7]; while if two synergies are considered, the activity of the trunk muscles and the tibialis anterior is divided into two distinct synergies in which one controls the trunk position and the other controls the foot movement [4,8,9]. Several studies examined muscle synergies in healthy participants in different conditions, such as at different speeds, in the shift from walking to running, and on curvilinear trajectories, and these synergies were shown to be robust across individuals and walking conditions [10], such as changes in walking speed [8,11], body weight support [4] or shifting from walking to running [12]. In pathology, the number of extracted synergies is, in general, lower and depends on the severity of the disease [13]. Stroke patients have less synergies on the paretic side and synergy composition becomes less selective and less sparse, indicating increased co-activation patterns [14,15,16]. Indeed, modules extracted from healthy controls can merge into only two or three modules, reducing the complexity of motor control. Patients with cerebral palsy (CP) showed fewer synergies with respect to healthy controls, similar to stroke patients, indicating the use of a simplified motor strategy compared to healthy people [17].

While representing the coordinated neural input to muscles (muscle synergies) and the coordinated joint motor output (kinematic synergies), conventional approaches, such as NMF or PCA, may fail in finding a direct, quantifiable link between the neural drive and the motor output they generate. Indeed, these conventional approaches have some limitations, such as the constraint of non-negativity for the NMF, which does not allow for modeling unconstrained data (such as kinematics) that can be positive or negative, indicating joint flexion or extension [18]. PCA may capture this type of data, but the constraint of orthogonality between the principal components makes this method not ideal for capturing the link with task space, as no physiological observation supports the hypothesis of orthogonality between the extracted synergies. It follows that synergistic analyses have been mostly based on single domains. The investigations so far assessed muscle synergies and kinematic synergies separately. When some approaches tried to combine the two domains, they were usually based on qualitative or separate assessments. Indeed, biomechanical functions were related to synergies only on the base of the correlation of temporal activations during the gait cycle, but no direct combination between muscle synergies and kinematic outputs was performed [19]. However, the neural and motor output components were not integrated into a single model. The recently designed mixed matrix factorization (MMF) allows the factorization of any combination of positively constrained data (such as processed EMG envelopes) and unconstrained data (such as kinematics data, which can either be positive or negative) and finds common activations between the two domains in a unique set of kinematic–muscular synergies [18,20]. Kinematic–muscular synergies, that incorporate both the muscular activation and the functional joint output, provide a more comprehensive, biomechanically oriented characterization of motor control. MMF was already applied to study hand coordination, showing that more synergies are needed when both muscle and kinematic data are used to achieve the same reconstruction accuracy and that muscle activations can be related to different biomechanical functions [21]. Since in gait analysis, muscle synergies can usually be related to gait phases and, therefore, to biomechanical functions, gait is one natural field of application for kinematic–muscular synergies. Several limitations affect current analyses made with synergistic approaches so far. First, a limited number of muscles is analyzed for each leg (typically, eight muscles [22]). Second, the available studies may provide a limited biomechanical interpretation: some synergies might show multiple peaks in temporal components that are associated with different task space functions, that are not captured and described clearly with standard muscle (or kinematic) synergies. Moreover, the role of some synergies may be equivocated as their functional role can be ambiguous (e.g., a synergy might be related to agonist action or to co-contraction to increase limb stiffness). Such effects could be particularly emphasized in pathology, where the mapping between muscle activations and motor output is not trivial [23].

Following this rationale, the primary aim of this study is to prove the feasibility and the added value of extracting kinematic–muscular synergies with respect to muscle synergies. Indeed, we wanted to demonstrate that kinematic–muscular synergies incorporate a more detailed description than muscle synergies alone when extracting the same number of muscle and kinematic–muscular synergies without altering the muscular part. A secondary objective is to show that a greater number of kinematic–muscular synergies are needed than muscle synergies to achieve the same reconstruction accuracy. Indeed, kinematic–muscular synergies embed a functional role into synergy weights and separate synergies that have the same structure but provide different biomechanical functions. 

## 2. Materials and Methods

### 2.1. Participants

Data from fifteen subjects (7M, 8F; 23.8 (2.1) years; height: 1.69 (0.11) m; weight: 66.6 (10.8) kg) were considered for this study. Data are from a publicly available dataset recently published [24], which includes healthy subjects, with no neurological or musculoskeletal impairments.

### 2.2. OpenSim Model and Analysis Pipeline

**Figure 1:**
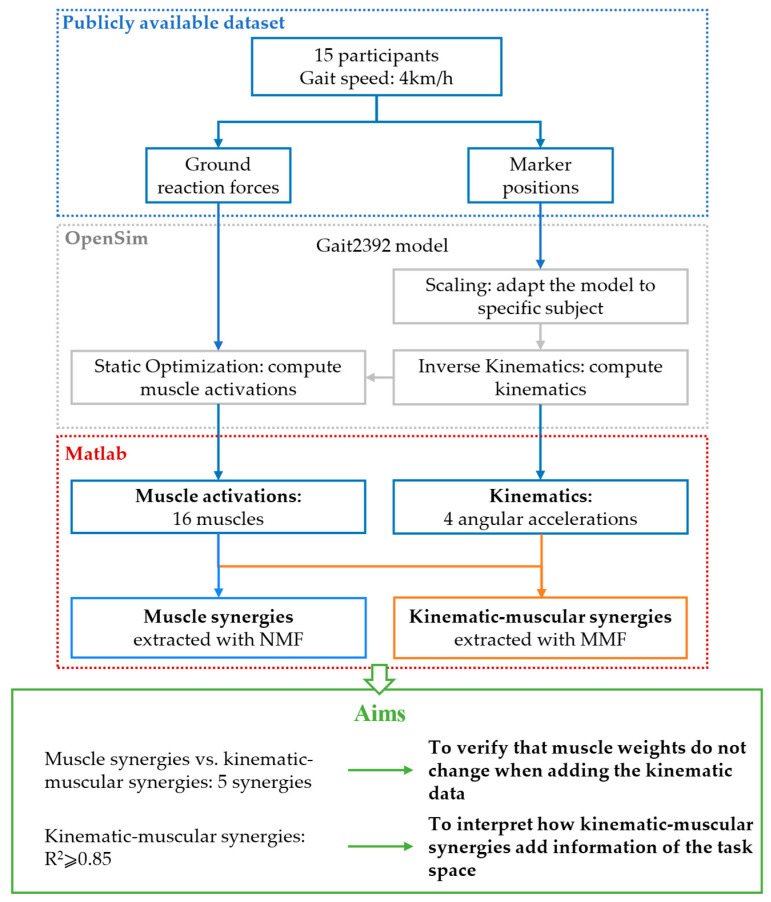
Scheme of the work. Markers’ position and ground reaction forces from a publicly available dataset are used as input for musculoskeletal simulations in OpenSim. The outputs of the model are kinematics and muscle activations. In total, 16 muscle activations are used for extracting muscle synergies with NMF and the same muscle activations with 4 angular accelerations are used for extracting kinematic–muscular synergies with MMF. Then, five kinematic–muscular synergies are compared to five muscle synergies to demonstrate that the muscular weights do not change when adding kinematic data. Finally, a number of kinematic–muscular synergies achieving R^2^ ≥ 0.85 are extracted to show that they add information from the task space.

Musculoskeletal simulations were performed in OpenSim v4.4 [25], using the available 3D Gait2392 model that simulates human gait [26,27]. The model includes 23 degrees of freedom of trunk and lower limbs and 76 muscles of the lower limbs and trunk. Twenty-four markers were placed in the model to match the motion analysis assessment protocol followed by Moreira et al. [24]. Marker trajectories and ground reaction forces (GRFs) were provided with a sample frequency of 200 Hz. The dataset includes seven different gait speeds; however, for this study, we considered only trials at 4 km/h, which is a comfortable natural walking speed. First of all, the OpenSim model was scaled to meet each participant’s anthropometry. Then, joint kinematics was computed with the inverse kinematics tool, giving the 3D marker trajectories as input to the OpenSim model [28]. Muscle forces were computed with the static optimization procedure provided in OpenSim [27], starting from the resulting joint kinematics and the GRFs. Muscle forces are calculated minimizing the instantaneous total square muscle activations needed to achieve the experimentally acquired trajectory and the model includes the muscle force–length–velocity relationships. Four strides performed with the right limb were considered for the analysis of each subject. The events for segmenting each stride (begin and end sample of each stride) were defined as two consecutive contacts of the right heel with the ground and they were identified based on the GRFs. Articular angles and muscle activations were filtered with a 3rd-order Butterworth low-pass filter with a cut-off frequency of 6 Hz. The degrees of freedom considered for the analysis were the pelvis flexion, the hip flexion, the knee flexion, and the ankle flexion in the sagittal plane. The angular acceleration for each joint was computed as the second derivative of the articular angles. Sixteen muscles often used in muscle synergy analysis were considered: Soleus, Gastrocnemius medialis, Gastrocnemius lateralis, Tibialis anterior, Vastus medialis, Vastus lateralis, Rectus femoris, Biceps femoris long head, Biceps femoris short head, Semimembranosus, Semitendinosus, Tensor fascia latae, Gluteus medius, Gluteus maximum, Adductor longus, Psoas. Both joint accelerations and muscle activations were segmented into strides, and they were resampled at 101 samples for each stride. Data were rearranged in a matrix for synergy extraction. For extracting muscle synergies, the matrix data had 16 rows, one for each muscle, and 404 columns, corresponding to 4 strides of 101 samples (per subject). For extracting kinematic–muscular synergies, the matrix data had 20 rows, representing 4 joint accelerations and 16 muscles, and 404 columns, corresponding to 4 strides of 101 samples. To allow inter-subject comparisons, kinematic data from each degree of freedom and EMG data from each muscle were normalized by the maximum found in all strides for each subject. The scheme summarizing the pipeline of the study is shown in Figure 1.

### 2.3. Synergy Extraction and Clustering

Methods for synergy extraction decompose the input signal ***x***(*t*) (generally the EMG) as the product of *n* time-invariant synergy vectors ***w_i_*** shared across all stride repetition and corresponding time-varying activation coefficients *c_i_*(*t*) specific for each stride repetition, as follows:

(1) $$x\left(t\right)=\sum_{i=1}^{n}w_{i}c_{i}\left(t\right)$$

The algorithm iteratively decomposes ***x*** to minimize the error between the original signal and the reconstructed signal, obtained from the product between the synergies and the temporal coefficients. Spatial synergies and temporal coefficients were extracted from data of each subject with two algorithms. The NMF iterative algorithm based on multiplicative updates was used for muscle synergy extraction, giving the EMG signals as input ***x*** [29]. The MMF algorithm was used for kinematic–muscular synergy extraction, giving the EMG and kinematics signals as input ***x*** [18]. MMF extends the standard NMF, removing the constraint of non-negativity of signals to be factorized (and extracted), and is based on a gradient descent update rule. For the MMF algorithm, we chose the following set-up values: λ = 200 and μ = 0.05. These parameters are selected because they offer a good trade-off between an accurate EMG reconstruction and fast algorithm execution [18]. The quality of reconstruction R^2^ of the original signal was defined for both the extractions as 1 − SSE/SST, where SSE is the sum of the squared residuals and SST is the sum of the squared differences with the mean input vector [30]. The algorithm was repeated 20 times and the solution achieving the highest R^2^ was considered for the analysis. The procedure was performed increasing the number of extracted synergies in the factorization from 1 to 16 for muscle synergies and from 1 to 20 for kinematic–muscular synergies. A linear mixed-effects model was fitted in order to assess the differences between the R^2^ obtained for muscle and kinematic–muscular synergies [31]. First, both R^2^ were tested for normality at each number of synergies using the Kolmogorov–Smirnov test. Then, the R^2^ was modelled as follows: *R*^2^ ∼ 1 + *n_syn_*·*model* + (1|*subject*) where model (*muscle/kinematic muscular*) and *n_syn_* (number of extracted synergies) were fixed effects with interaction, *n_syn_* was a categorical variable, and subjects were included as random effects on intercept. The level of significance (*α*) was set at 0.05.

Five muscle synergies were extracted for each subject since it is the higher number of synergies typically extracted from one subject in gait analysis [4]. In the first step of the analysis, the same number of kinematic–muscular synergies were extracted to allow direct comparison between muscle and kinematic–muscular synergies. For each participant, muscle and kinematic–muscular synergies were matched for similarity, computed as cosine angle between pairs of matched synergies. To perform this comparison, the muscle weights of the kinematic–muscular synergies were re-normalized to have unit norm and then they were matched for similarity with the paired muscle synergies.

Moreover, observing that five muscle synergies achieved R^2^ ≥ 0.85, in the second part of our analysis we extracted a number of kinematic–muscular synergies that achieved the same threshold to provide an assessment based on the same reconstruction accuracy.

Synergies from all participants were grouped with k-means clustering algorithm to reduce them to a small set of synergies shared by subjects that represent the repertoire of synergies available to healthy people and evaluate their variability [32]. The clustering procedure was repeated 100 times with new initial random cluster centroid estimates with the same number of clusters and the result with the lowest sum of Euclidean distances of each element in the cluster to the centroid was selected. This pipeline was repeated until the average inter-cluster similarity was greater than 0.70 in order to guarantee a limited number of clusters and a good intra-cluster similarity level [33,34]. As a measure of the robustness of the clustering, the intra-cluster similarity was computed with the cosine angle comparing all pairs of synergies in a cluster [35]. Five clusters were defined for muscle synergies and the same number of clusters were used to compare the kinematic–muscular synergies when extracting 5 synergies. Seven clusters were needed instead when extracting 6 kinematic–muscular synergies per subject.

## 3. Results

**Figure 2:**
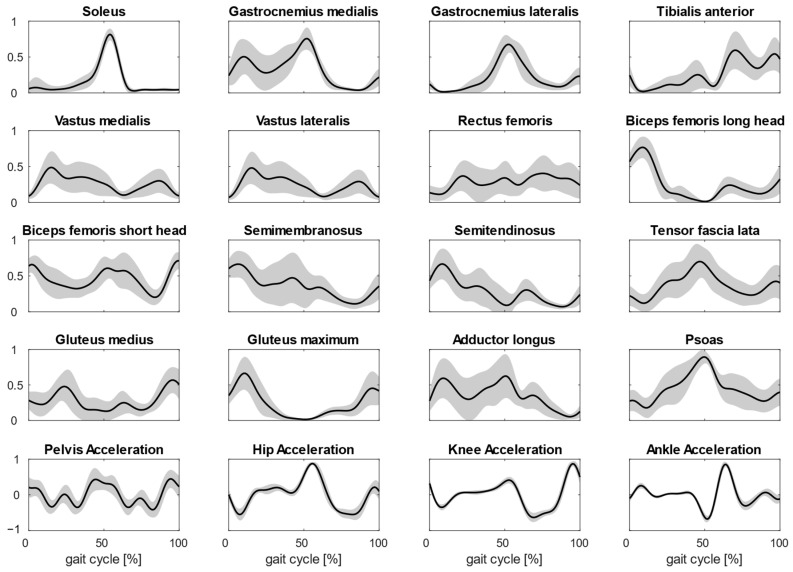
Plots show the averaged normalized activations of the 16 muscles considered during gait. The muscle activations are averaged on four steps and for all subjects. In the last row, joint accelerations used for MMF are shown too.

In Figure 2, the averaged muscle activations from the 15 subjects are shown (average values and deviations).

### 3.1. Reconstruction R^2^

**Figure 3:**
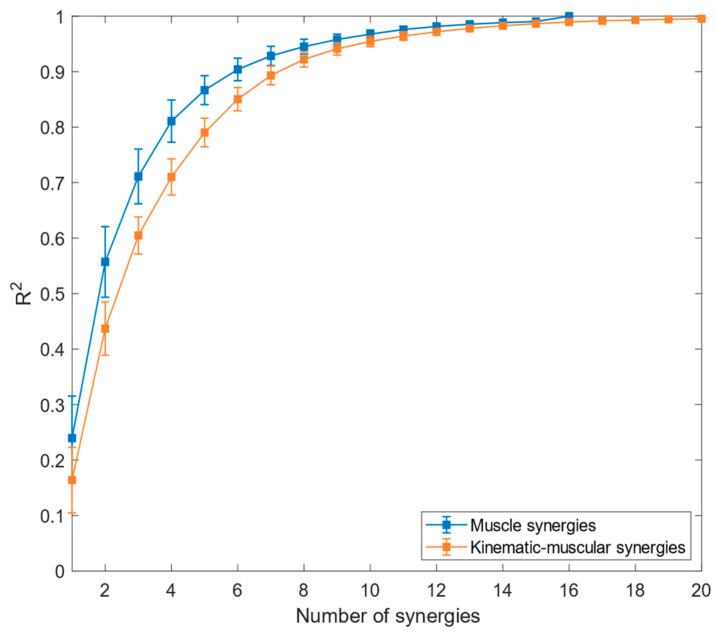
Reconstruction R^2^ for muscle synergies (blue graph) and kinematic–muscular synergies (orange graph). Means and standard deviations across subjects are reported.

The mean reconstruction R^2^ across participants is reported in Figure 3 for kinematic–muscular synergies and muscle synergies.

Referring to Figure 3, the reconstruction of R^2^ for muscle synergies has higher values than the R^2^ for the kinematic–muscular synergies. Moreover, the R^2^ for the kinematic–muscular synergies requires more synergies to reach R^2^ = 1 because the MMF includes 20 channels instead of 16. The linear-mixed effect analysis showed that model type (*muscle/kinematic–muscular*) has significant effects on R^2^ from 1 (*p* < 0.001, β = −0.065) to 7 (*p* = 0.03, β = −0.025). In the first part of the experiment, the R^2^ threshold to select the number of synergies in the muscle and in the kinematic–muscular configurations was set = 0.85; five muscle synergies were sufficient to reach this threshold, while for the kinematic–muscular synergies, six synergies were needed to reach the threshold.

### 3.2. Comparison between Kinematic–Muscular Synergies and Muscle Synergies

To highlight the first characteristics of kinematic–muscular synergies, in this study, we compared the muscle weights of muscle synergies and kinematic–muscular synergies. Their mean similarity is reported in Table 1. The similarity between the muscle weights of the models was consistently higher than 0.80 for all subjects and was significantly higher than the similarity between randomly paired synergies (*p* < 0.001). These results indicated that the muscular part is only minimally affected when extracting kinematic–muscular synergies. Thus, adding kinematic weights only minimally modified the muscle synergies extracted with NMF.

**Figure 4:**
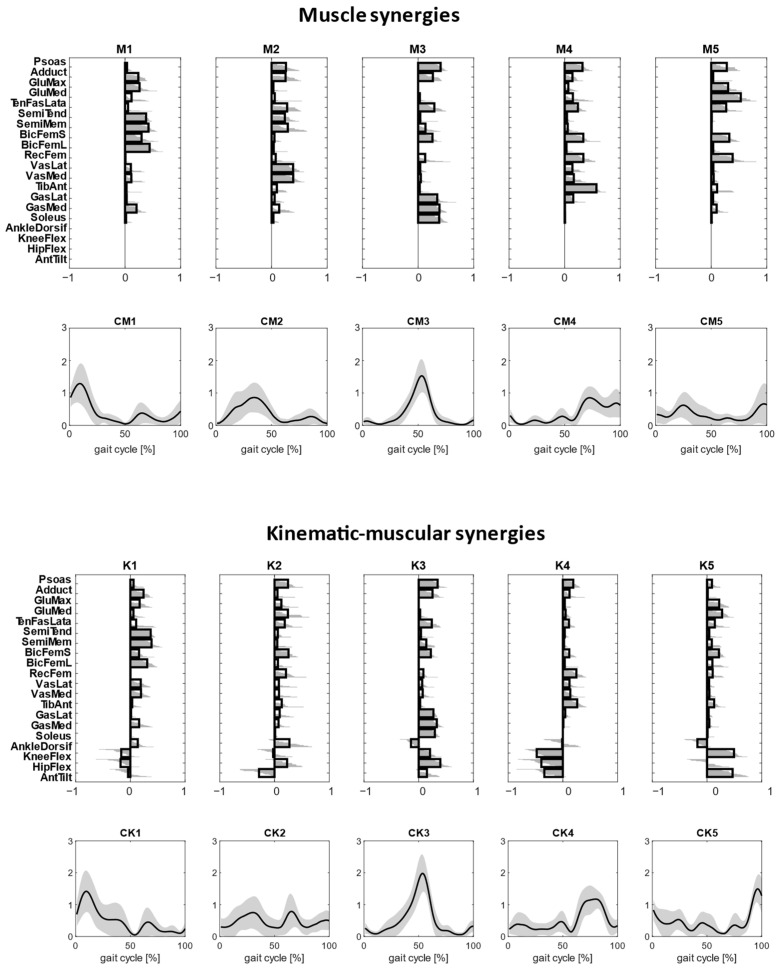
Clustered muscle synergies and corresponding temporal coefficients are reported in the top first panel. Clustered kinematic–muscular synergies and corresponding temporal coefficients are reported in the lower panel. Clusters are ordered based on synergy recruitment timings in the gait cycle.

Clustered muscle synergies and kinematic–muscular synergies were paired by similarity and are shown in Figure 4. All the kinematic–muscular synergies include weights that indicate which joints were accelerated due to muscle activity and could clarify whether each joint acceleration contributed to flexing or extending the joints. Referring to Figure 4, and denoting as M_i_ the ith muscle synergy and K_i_ the ith kinematic–muscular synergy, M1 and K1 were active at the beginning of the stance phase and show the activation of the gluteus and the hamstrings muscles, which extend the hip, and the activation of the vastus medialis and the lateralis that extend the knee as shown in kinematic–muscular synergies. M2 and K2 are active during the stance phase. M2 is characterized by the activation of the adductor, psoas, hamstring muscles and vastus medialis and lateralis. The kinematic–muscular synergy K2, instead, recruits many muscles with small activations, including the psoas, biceps femoris, rectus femoris. These muscles contribute to the flexing of the hip and to the posterior movement of the pelvis. M3 and K3 represent the push off of the gait cycle and are characterized by the psoas and the adductor, which flex the hip, and the gastrocnemii and the soleus that perform plantarflexion of the ankle and the flex of the knee. M4 and K4 are active during the swing phase and show tibialis anterior, psoas, biceps femoris, rectus femoris that extend the knee and the hip. The kinematic–muscular synergy K4 shows a higher magnitude of kinematic weights, reducing the muscle weights, probably as the limb is exploiting previous activation to perform the swing phase. Finally, M5 and K5 are active at the end of the gait cycle and are characterized by the activation of psoas, glutei and biceps femoris, flexing the knee and tilting the pelvis anteriorly.

### 3.3. Extraction of Kinematic–Muscular Synergies with R^2^ > 0.85

**Figure 5:**
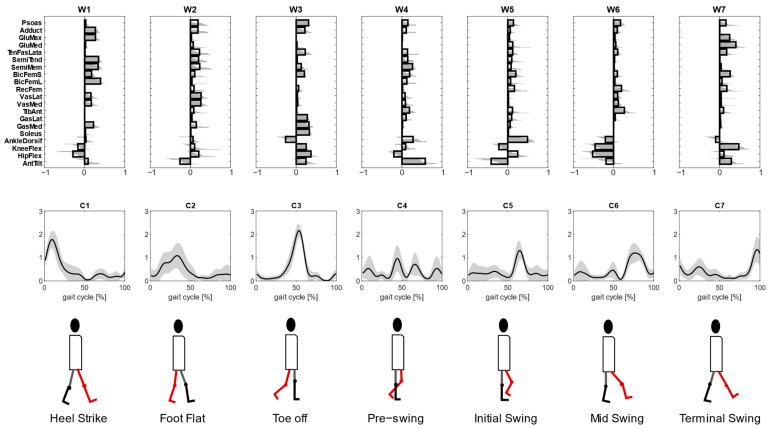
Kinematic–muscular synergies extracted with R^2^ ≥ 0.85 were grouped into 7 clusters so that the intra-cluster similarity is greater than 0.70 for all clusters (upper panel). The synergies activation coefficients are ordered following the gait cycle (lower panel). The third line represents the biomechanical function associated with the walking task.

To investigate the second characteristics of kinematic–muscular synergies, the study quantifies the assessments provided with kinematic–muscular synergies when their number is not equal to the number of muscle synergies but selected with a fixed R^2^ threshold (R^2^ > 0.85), as is usually carried out in experimental studies. We noted that the number of kinematic–muscular synergies required to reach the R^2^ threshold was six. Thus, more kinematic–muscular synergies were needed than muscle synergies to obtain the same level of reconstruction achieved with muscle synergies. Figure 5 shows the result of the clustering procedure applied to the whole dataset of the extracted kinematic–muscular synergies when R^2^ > 0.85. Seven clusters were found. They are presented in Figure 5 in chronological order following synergy recruitment in the gait cycle and denoted with a W_i_ label. The first synergy cluster W1 is associated with the beginning of the stance phase and the synergy cluster W7 is related to the end of the stride when the leg is repositioned on the ground before the new gait cycle.

Each cluster can be associated with biomechanical functionality within the walking task, depending on the muscles recruited, on the moving joints, and on the timings of activation. W1 shows a strong activation of the hamstring muscles accomplished with an activation of the adductor longus and the gluteus maximum that are responsible for hip extension and then shows the activation of the vastus medialis and the lateralis for knee extension. The stance phase continues with synergy W2 where an overall activation of many muscles with an initial posterior pelvic tilt can be observed. Then, W3 shows a strong activation of the soleus, gastrocnemius lateralis and medialis, which represent the part of the gait cycle in which the ankle is dorsiflexed, and the knee and hip are flexed during the late stance to provide propulsion to begin the swing phase and advance. Synergy W4 is activated just before the push off and at the beginning of the swing phase and shows the anterior pelvic tilt and the activations of the semitendinosus, semimembranosus, tensor fascia latae, biceps femoris and tibialis anterior. The swing phase is described in W5 where the pelvis changes its position, activating with a posterior tilt and the ankle is dorsiflexed. These kinematic activations are associated with both hamstring muscles, both biceps femoris, psoas and rectus femoris. The swing phase continues with full extension of all joints enabled by small activations of many muscles (W6). Finally, the gait cycle finishes with a knee flexion and anterior pelvic tilt that are needed to position the foot on the ground before a new gait cycle, as can be observed in synergy W7. In this synergy, the muscular part is characterized by a strong activation of the gluteus maximum and medialis, tensor fascia latae and short bicep femoris.

## 4. Discussion

### 4.1. Summary of the Findings

In this study, kinematic–muscular synergies were extracted with MMF during the locomotion of 15 participants for the first time. Kinematic–muscular synergies were compared to standard muscle synergies extracted with NMF. Gait data from a publicly available dataset were used to feed a musculoskeletal model in OpenSim, in which kinematics and muscle activations were computed. Muscle synergies were extracted from the activation of 16 muscles of the right lower limb with the standard NMF. Kinematic–muscular synergies were extracted from the same EMG activations and from the angular acceleration of four joints in the sagittal plane with the MMF algorithm. For a given number of extracted synergies, the reconstruction R^2^ was higher for muscle synergies with respect to kinematic–muscular synergies and fewer synergies were needed to achieve R^2^ > 0.85. Comparing the muscular part of the kinematic–muscular synergies to muscle synergies, the similarity was high for all the participants and significantly higher than the random similarity. This result showed that the muscular weights are minimally affected when adding the kinematic weights. Lastly, when a given R^2^ is selected, more kinematic–muscular synergies are needed, and this effect is linked to a more accurate, functionally oriented description of synergistic control. Thus, including kinematics in the synergy analysis allowed us to highlight the link between muscle activation and their biomechanical function, which was described by individuating, for the first time, the repertoire of kinematic–muscular synergies available to healthy people.

### 4.2. Muscle Synergies vs. Kinematic–Muscular Synergies

All participants showed good similarity between muscle weights of the kinematic–muscle synergies and muscle synergies. This finding confirmed previous results [18] that showed that the addition of kinematic weights when using the MMF algorithm does not alter (or minimally alters, in the case of noisy data) the composition of standard muscle synergies originating from the neural structures. Indeed, muscle synergies and the muscle weights of kinematic–muscular synergies were mostly highly similar, and the main differences were found in M2 and K2 during the stance phase. In each cluster, the kinematic–muscular synergies add information on how joints move and associate it to standard muscle synergies. For example, gastrocnemii and soleus plantar flex the ankle, hamstring muscles flex the knee and extend the hip, while psoas, rectus femoris and vastus medialis and lateralis flex the hip. The differences between muscular and kinematic–muscular synergies may be related to the fixed chosen number of synergies. Indeed, the kinematic–muscular synergy model needs more synergies to reach the same reconstruction accuracy as the muscle synergy model and, therefore, five synergies are not enough to describe the biomechanics of gait in detail. In fact, when extracting six synergies, clusters W1, W2, W3, W6 and W7 are very similar to the muscle synergy clusters of Figure 4. The two added synergies, W4 and W5, describe the transition from the stance to the swing phase during the gait cycle. In particular, in W4, all muscles are involved in body stabilization, during ankle dorsiflexion and anterior pelvic tilt that characterize the exploitation of the propulsion produced in the late stance. W5, instead, represents the transition from back tilt to front tilt needed to prepare the body to heel strike the homolateral foot.

### 4.3. Kinematic–Muscular Synergies Add Functional Information to Muscle Synergies

With kinematic–muscular synergies, muscle activity can be associated with the kinematic accelerations that result from muscle contraction, enriching standard neural synergies with a functional role. Kinematic–muscular synergies incorporate task execution variables into muscle synergy extraction, providing a functional role to each synergy. In this way, functional synergies improve the interpretation of the results and their clinical use. Synergies from all the participants were clustered into seven mean synergies. Each synergy can be associated with a biomechanical functionality describing the phases of a stride depending on the muscles recruited and the timings of activation. Observing the kinematic part of kinematic–muscular synergies, one can unveil the functionality of each muscle module expanding the previous literature in which muscle or kinematic synergies were extracted. The first cluster of synergy M1, representing the early stance, shows a strong activation of the gluteus, tensor fascia latae, vastus medialis and lateralis, which are responsible for the stabilization of the hip joint during the heel strike and the load acceptance phase. In kinematic–muscular synergies (Figure 5), this gait phase was divided into two synergies: W1 with a strong activation of gluteus maximum and W2 with a strong activation of both tensor fascia latae and vastus lateralis muscles. In addition, in W1, there is also a high activation of semitendinosus and biceps femoris that contribute to the leg’s stabilization during movement. In these synergies, the kinematic weights are reduced, and this finding reflects how, in this stride phase, the neuromuscular system works more to prepare to accept the load rather than to carry out the movement. The forward propulsion is well represented both considering muscle and kinematic–muscular synergies, in fact, in both cases, the synergies of M3 and W3 highlight a strong activity of the soleus and the gastrocnemius. Moreover, observing the kinematic–muscular synergy W3, other muscles useful to generate a propel propulsion are the adductor and the psoas that help to maintain balance [36].

The initial swing phase is represented in different ways in the literature because it depends on the muscles considered in each study. In fact, according to some studies, this phase principally involved the trunk muscles, such as the longissimus dorsi or the erector spinae, because, during the initial swing phase, the trunk position needs to be controlled in the frontal plane at the time of contralateral foot heel strike and ipsilateral foot lift [8]. In our study, we did not monitor trunk muscle activation, but we coherently observed in synergy W4 an activation of all the muscles involved in body stabilization, with a slightly accentuated activation of the tibialis anterior, semimembranosus, short biceps femoris and also a moderate activation of psoas. Therefore, the muscular part of kinematic–muscular synergy W4 has a lower magnitude, but it gives a clear view of joint motion showing ankle dorsiflexion and a strong anterior pelvic tilt that characterize the exploitation of the propulsion produced in the late stance [37]. In the kinematic–muscular synergies, another synergy is added, W5, that represents the swing phase when the pelvis makes the transition from back tilt to front tilt [38]. Indeed, we observed the activation of the tensor fascia latae, both the hamstring muscles and both bicep femoris that are responsible for this transition of the legs linked to the change of pelvic position, and the kinematic part shows a strong posterior pelvic tilt and ankle dorsiflexion and moderate hip extension and knee flexion that are needed to prepare the body to heel strike the homolateral foot. In kinematic–muscular synergy W6, this function is well represented, since the same muscle activations and the kinematic parts show an extension of all the joints of the leg and a posterior pelvic tilt that are typical joint movements to allow the deceleration of the foot.

Finally, in the muscle synergy associated with the late swing phase, the hamstrings are the key muscles, as they decelerate the leg [39]. The kinematic–muscular synergy associated with this phase (W7) also shows the activation of the bicep femoris and the rectus femoris, but it especially highlights a strong activation of the gluteus medialis and maximum and tensor fascia latae. Moreover, the kinematic part of this synergy is well accentuated and shows a strong knee flexion that is needed to control and attenuate the load due to the leg repositioning before a new gait cycle.

Kinematic–muscular synergies were shown to be consistent with the muscle modules that are usually identified in the literature, such as the activation of the glutei, tensor fascia latae, vastus medialis and rectus femoris in the stance phase; gastrocnemii and soleus activated during the push off; rectus femoris and tibialis anterior activated in the swing phase; and the hamstring muscles at the end of the swing phase [4,7,8,9]. This means that the kinematic–muscular synergy extraction adds information about how the movement is performed without altering information about the muscle activations. As an advantage, not only are the muscle activations clearly associated with a functional role, but also a fractionation effect is shown that helps to provide a more accurate description of synergistic control. For example, W5 and W6 are synergies that refer mainly to the swing phase and show a high predominance of kinematic coefficients. This is expected as the swing phase exploits inertial forces generated in the stance and late stance phases and thus contains more motion rather than neural drive. Coherently, synergies like W2 and W3 that represent the stance and late stance phase show a mixture of muscle and kinematic weights as muscle activation is needed to generate propulsion during the gait cycle. The extraction of kinematic–muscular synergies allows us to directly integrate the neural activations and the motor output in an efficient way with respect to other approaches based on the correlation of muscle and kinematic synergies, as performed by Esmaeili et al. [19]. In fact, we presented a joint analysis in which muscular and kinematic weights are extracted together in multidomain synergies. Moreover, synergies are extracted from joint accelerations and not from joint position, allowing us to directly couple the muscle activity and the joint movement on which they act. In Esmaeili et al. [19], instead, kinematic synergies were extracted from the joint position. For instance, the effect of the glutei muscles of the first synergy was visible in the second synergy, characterized by hip extension; the activity of the calf muscles during the push off generated the ankle dorsiflexion in the following synergy. Therefore, kinematic–muscular synergies provide a straightforward analysis to associate muscular activity and kinematic output, directly defining the functionality of each synergy.

### 4.4. Clinical Application of Kinematic–Muscular Synergistic Control

The use of synergies in motor control assessment has diffused in recent years, but the current synergy-based analysis methods do not yet exploit the full potential of the synergistic approaches. In fact, the standard synergistic models have some limitations, such as the inability to consider the task space variables. Incorporating task execution variables into muscle synergy extraction links muscle synergies to the motor function they produce. This process reflects the task space output due to muscle synergies and improves the interpretation of the results and their clinical use [40]. The coupling between the two may help in fostering synergistic protocols as suggested in recent works [41,42]. Moreover, muscle synergy analysis is still scarcely used for evaluating neuromotor rehabilitation in clinical scenarios. However, it was shown that modifications in muscle synergies can measure the progression of the rehabilitation process in an interpretable and quantitative manner [43]. Further improvements in the standard synergy analysis are needed before it can be fully transferred to clinical practice. This forward step, which includes the task space into muscle synergy analysis, may improve the interpretability of the results and may help the introduction of synergy analysis in the clinical practice and clinical decisional process, providing clinicians and therapists with a novel instrument to assess the efficacy of a therapy analyzing movement kinematics associated to the underlying neural control strategies.

The first step for such applications is to provide reference databases of healthy people to create a repertoire of kinematic–muscular synergies and this study is a pilot investigation in such a sense. Using kinematic–muscle synergies may improve the understanding of neuromotor coordination in several diseases. The practical consequences of adding kinematic weights to synergies impact the understanding of how the underlying neural motor strategies are reflected at task space and biomechanical levels [18]. Therefore, functional synergies may open new perspectives in the analysis of motor control in clinical practice providing a more in-depth evaluation of pathological motor patterns, especially in patients with impaired control of movement coordination, as the kinematic–muscular synergies may link directly muscular and kinematic patterns allowing to elucidate the relationship between the neural drive and motor outcomes [21]. Some of the practical added values of including kinematic weights to standard muscle synergies are proposed below.

First, the exploitation of functional synergies can add significant details on the altered muscle synergies of patients who are affected by pathologies that compromise movement coordination. Altered coordination can be associated with an altered motor output in different ways. Indeed, the first achievement of kinematic–muscular synergies is to show if altered coordination is the result of altered synergies or if synergies similar to physiological ones are available but are recruited in a biomechanically abnormal way. Second, muscle synergies with similar compositions might be extracted from healthy and pathological subjects; however, their biomechanical functions can be associated with different joint movements, which is fully resolved and clarified only with kinematic–muscular synergies. In such a sense, kinematic–muscular synergies might be a very useful tool to highlight the biomechanical function associated with each neural synergy. Third, in standard muscle-synergy assessment, there might be some muscle synergies that present multiple peaks of activation within a movement phase; kinematic–muscular synergies might instead associate such muscle coactivation peaks to specific kinematics, with the effect of producing a fractionation of muscle synergies that leads to a more specific interpretation of their biomechanical function. Fourth, using kinematic–muscular synergies may set a novel target for rehabilitation: restoring not only physiological, “neural” synergies but also promoting their efficacy at the task level.

For instance, it was shown that the locomotion of post-stroke patients with severe impairment is characterized by the merging of muscle synergies and patterns may change depending on the level of impairment [15]. In some cases, this effect is coupled with a reduction of the joint range of motion with respect to physiological walking. Kinematic–muscular synergies would naturally capture the neural–motor output relationship by associating reduced kinematic coefficients to the functional synergies of patients with respect to healthy people. Thus, abnormal muscle couplings would be interpreted with the support of a direct link to their effect on the task space. Hence, these synergies’ responses manifesting at different levels of impairment in patients with stroke, may represent a precise and quantifiable marker of the physiological status of the patient in order to better understand the complex processes that follow accidents involving the cortical and spinal motor system and so guide the development of different rehabilitation approaches [16]. This result can be achieved by further classifying patients’ synergistic control not only on the basis of abnormal coactivations but also by considering the biomechanical outcomes that are produced by such coactivations.

In Parkinson’s disease (PD), motor disorders are the main symptoms that become the major source of disability with the progression of the disease and include tremors, bradykinesia and loss of balance. Several studies used muscle synergy analysis to characterize motor control in patients with Parkinson’s disease and they revealed a lower number of extracted synergies compared to healthy subjects [44]. Therefore, a reduced number of recruited muscle synergies may be a sign of an alteration in muscle activation patterns that could explain changes in motor control in patients affected by neurological disorders [45]. These altered muscle activation patterns are often associated with a modified kinematic movement; by extracting kinematic–muscular synergies, investigators could better highlight the motor alterations due to motor disorders such as tremors and bradykinesia. Moreover, when analyzing postural control in patients with Parkinson’s disease, it was noted that they are characterized by smaller indices of anticipatory synergy adjustments compared to healthy subjects because patients are affected by freezing of gait [46]. This symptom should be better investigated by observing the kinematic–muscular synergies that link the muscle activation patterns to the joint activated during motor movements so that researchers better understand how an alteration at the neural level is reflected in an alteration at the motor level.

Another pathological condition that affects movement is Spinal Cord Injury (SCI). Muscle synergies have been used in order to understand how the spinal circuitry reorganization after spinal cord injury reflects also in the modular organization [47]. SCI patients show significantly altered muscle synergy patterns [48,49] and directly linking the kinematic output to neural synergy alteration may shed light on functional recovery and neural plasticity. Moreover, it was found that despite consistent muscle synergies being extracted across SCI patients in upper limb movements, this outcome was coupled with a high level of variability in kinematic strategies [50]. Kinematic–muscular synergy analysis seems the natural choice to investigate this result and understand how similar muscle synergies may map into different joint movements.

Children with Cerebral Palsy (CP) show abnormal motor control resulting in altered coordination and stiff muscles. It was shown that locomotion is altered with different degrees of severity depending on the functional impairment, strength and presence of spasticity [17]. Usually, fewer muscle synergies are recruited by CP patients with respect to healthy people, with some abnormal synergies specific to the CP [22]. Moreover, wider temporal activation patterns are found in children with CP [51]. Synergy variability is high between CP children and synergy structure is more altered in patients with greater impairment [23]. However, providing a link between the muscle activation and the motor output is not always trivial [52] and kinematic–muscular synergies may support the understanding of pathological movement. The proposed method provides a direct link between the underlying neurophysiological mechanisms and their motor output, thus fostering the understanding of the progression of the pathology, the consequent choices regarding the selection of the best rehabilitation course, and the evaluation of the effects of surgical interventions.

The restoration of motor output is usually the primary objective of motor rehabilitation and approaches based on kinematic–muscular synergies can be used to implement individualized therapeutic strategies promoting a general restoration of motor control. It is demonstrated that muscle and kinematic synergies can be modified by targeted motor training developed using assistive approaches, such as functional electrical stimulation (FES) which are potentially useful to re-establish the normal ways of muscle activation that was impaired by neurological disease [53]. FES was developed to design a rehabilitation therapy based on muscle synergies because if the muscle stimulation reflects the healthy muscle activation the neuroplasticity may reorganize the neural circuitry of motor control to restore a normal pattern of muscle activation [54]. Kinematic–muscular synergies can be used to better customize FES therapy trying to make the therapies also task-specific so that the rehabilitation therapy becomes more focused on restoring specific motor control [55]. Furthermore, in rehabilitation, robotic devices and exoskeletons may be used to assist locomotion in patients with hemiparesis in order to elicit the restoration of motor control. Exoskeletons are robotic interactive wearables that actively assist motor-impaired individuals during walking and adjust abnormal gait by providing suitable assistive forces [56]. Muscle and kinematic synergies have been used not only for evaluating the effects of using exoskeletons on motor control but also for adjusting the level of assistance of the paretic limb based on the non-paretic limb synergies aiming at increasing the similarity between synergies [57,58]. Therefore, kinematic–muscular synergies provide a comprehensive evaluation of motor control, fusing both neural and kinematic information that can be used for assessing the restoration of motor control when using exoskeletons and improving the design of these devices.

## 5. Limitations and Future Work

Although the analytical approach and the potential applications proposed in this work are novel, the EMG data used were computed based on an OpenSim simulation starting from experimental kinematic data. Such simulated EMG data may be only partially representative of the real data and further work should be performed on the experimental data. Moreover, experimental input data were taken from healthy participants only. Thus, it would be interesting to investigate how this approach improves the understanding of the pathophysiology of several neurological disorders associated with motor control impairments, such as stroke, Parkinson’s disease or cerebral palsy, and to study to what extent healthy people’s control strategies are preserved in neurological patients and which novel insights can be obtained with kinematic–muscular synergies.

## 6. Conclusions

In this study, for the first time, functional kinematic–muscle synergies were extracted with the novel mixed matrix factorization from locomotion data. Data from 15 participants from a publicly available dataset were used to feed a musculoskeletal model in OpenSim used to compute lower limb muscle activations, muscle synergies and kinematic muscular synergies. We demonstrated that kinematic–muscle synergies can describe the biomechanics of motion to a better extent than muscle synergies alone and are increased in number to account for the different biomechanical roles that muscles have within a movement. Despite only healthy subjects being investigated in this study, the results suggest that applying this approach to patients could also be beneficial in improving the understanding of the pathophysiology of neurological disorders related to motor impairment. Therefore, this approach may have an impact on future work in improving the understanding of pathologies in rehabilitation.

## Figures and Tables

**Table 1:** Mean similarities between matched muscle and kinematic–muscular synergies are reported for all subjects. Standard deviations are reported in brackets.

Similarity between Matched Muscle and Kinematic–Muscular Synergies		
Subject	Mean	Random Similarity
S01	0.898 (0.141)	0.493 (0.276)
S02	0.881 (0.132)	0.507 (0.279)
S03	0.824 (0.154)	0.465 (0.259)
S04	0.970 (0.029)	0.532 (0.251)
S05	0.823 (0.245)	0.422 (0.299)
S06	0.878 (0.116)	0.488 (0.257)
S07	0.926 (0.094)	0.532 (0.245)
S08	0.939 (0.073)	0.487 (0.283)
S09	0.809 (0.251)	0.489 (0.263)
S10	0.868 (0.170)	0.470 (0.283)
S11	0.908 (0.069)	0.547 (0.255)
S12	0.845 (0.062)	0.510 (0.264)
S13	0.878 (0.152)	0.523 (0.264)
S14	0.873 (0.169)	0.516 (0.253)
S15	0.889 (0.217)	0.519 (0.255)
**Total**	**0.881 (0.045)**	**0.500 (0.032)**

## Data Availability

Processed data are available from the corresponding author upon reasonable request.
